# Beyond Working Memory Capacity: Attention Control as the Underlying Mechanism of Cognitive Abilities

**DOI:** 10.3390/jintelligence14020022

**Published:** 2026-02-02

**Authors:** Yoonsang Lee, Randall Engle

**Affiliations:** School of Psychology, Georgia Institute of Technology, Atlanta, GA 30332, USA; randall.engle@gatech.edu

**Keywords:** attention control, working memory capacity, executive attention, fluid intelligence, interference control, individual differences, latent-variable modeling, cognitive measurement

## Abstract

Working memory capacity (WMC) has long served as a central indicator of individual differences in complex cognition. However, growing evidence suggests that a substantial portion of its predictive power may reflect attention control (AC)—including goal maintenance, interference management, and inhibition—rather than storage capacity alone. This review synthesizes findings across six domains: (1) perception and sensory discrimination, (2) learning and problem solving, (3) cognitive control and decision making, (4) retrieval and memory performance, (5) multitasking and real-world performance, and (6) clinical applications. Across these areas, WMC-related effects frequently align with demands on AC, though the strength and nature of this alignment vary by domain. We highlight the importance of incorporating reliable AC measures and recommend latent-variable approaches to more clearly separate storage, control, and representational processes underlying complex performance.

## 1. Introduction

For decades, working memory capacity (WMC)—the limited amount of information that can be actively maintained under cognitive load without rehearsal (Wilhelm et al., 2013)—has been shown to be a key factor affecting performance in complex cognitive tasks. Historically, the concept of working memory evolved from earlier models of short-term memory, which emphasized storage of information (Atkinson & Shiffrin, 1968). These models proposed short-term and long-term stores with limited temporary storage, where information was maintained mainly through rehearsal processes. However, these structural approaches failed to account for the complex and dynamic nature of memory performance in demanding cognitive tasks (Engle & Oransky, 1999). To address this, A. D. Baddeley and Hitch (1974) proposed a model of working memory, which implies a system that combines storage with active manipulation, known as the central executive (A. Baddeley, 2000, 2012). This domain-general control system regulates attention and coordinates subsystems that manage domain-specific representations. This shift in emphasis—from passive storage to executive control—reframed working memory as a mechanism for attentional regulation and coordination, attributing it to performance across various domains.

While WMC has often been treated as a unified capacity, researchers have long debated whether its predictive power reflects discrete short-term storage mechanisms (e.g., binding, refreshing) or top-down executive control processes. Some models emphasize domain-specific storage buffers (Logie, 2011), others propose capacity limits arise from interference-prone binding (Oberauer, 2009), and still others focus on the role of attention in actively maintaining representations (Engle, 2002; Cowan, 2011).

This diversity of views raises a central question: To what extent is WMC a distinct cognitive construct, and to what extent is it best understood as reflecting more fundamental AC mechanisms?

### 1.1. Attention Control as the Shared Mechanism of Working Memory and Fluid Intelligence

Attention control (AC) is referred to as the domain-general ability to operate information processing in service of current goals (Engle, 2002; Shipstead et al., 2016). It enables individuals to maintain goal-relevant representations, resist interference, and disengage from outdated or irrelevant content (Oberauer, 2024). AC is not a unitary construct but comprises at least three subfunctions: goal maintenance, interference resolution, and disengagement (Burgoyne & Engle, 2020; Engle & Kane, 2004). These components support top-down regulation of attention across a wide variety of cognitive contexts.

This review is grounded in the executive attention framework, which holds that individual differences in WMC (WMC) primarily reflect attention control mechanisms—rather than passive storage (Engle, 2002). According to this view, WMC tasks index the efficiency of AC—specifically, the ability to maintain relevant information and suppress interference under dual-task demands. This reinterpretation gained empirical support from latent-variable modeling studies. Engle et al. (1999) first introduced this view, revealing that WMC, unlike short-term memory, shares variance specifically with fluid intelligence (gF) due to its attentional component through latent analysis approach. Engle (2002) further demonstrated that the link between WMC and gF is driven by AC processes, especially sustaining focus and resisting distraction. Subsequent research has consistently supported and expanded upon this attention-centric view (e.g., Draheim et al., 2022; Heitz et al., 2005; Shipstead et al., 2015).

Notably, the executive attention view is not the only interpretation of WMC. Other accounts have emphasized domain-specific storage (Logie, 2011), context-sensitive binding (Oberauer, 2009), or strategic retrieval from secondary memory (Unsworth & Engle, 2007). While these perspectives differ in emphasis, the mechanisms they invoke—selecting among bound representations, maintaining contextual associations, or guiding memory search—are themselves often theorized to depend on goal-directed control. Rather than opposing the WMC as an AC framework, they offer complementary insights into the representational systems upon which attention operates. In this review, we focus on attention control as the organizing constraint that enables or regulates these lower-level mechanisms.

Fluid intelligence (gF) also occupies a central position in this framework. Across hierarchical ability models, gF is defined as the capacity to perform deliberate, effortful reasoning on novel problems—drawing inferences, integrating relations, and constructing new representations (Ackerman & Heggestad, 1997; Kyllonen & Christal, 1990; Primi et al., 2010). These demands rely on controlled processing rather than prior knowledge, which is why gF consistently correlates with executive functions. Reasoning tasks require coordinating attention across intermediate representations, suppressing misleading or habitual responses, and revising partial solutions when they no longer support progress (Duncan et al., 2012; García-Madruga et al., 2022). Although WMC and gF are strongly related, alternative models—storage-based (Chuderski, 2014), relational-integration (Oberauer et al., 2008), and executive-attention (Engle & Kane, 2004)—converge on the idea that both depend on goal-directed control. In this view, WMC reflects the ability to maintain stable, interference-resistant representations, whereas gF reflects the ability to flexibly reorganize or update them in response to novelty.

**Figure 1:**
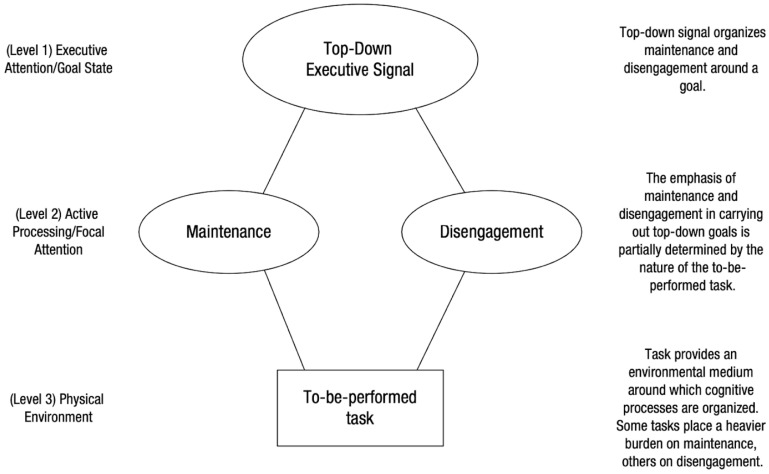
Schematic illustration of the maintenance and disengagement components described in Shipstead et al. (2016). The diagram depicts two proposed control processes—maintenance and disengagement—organized under a broader attentional-control framework.

A key mechanistic implication of this framework is that WMC and gF place differential demands on distinct subfunctions of attention control. Shipstead et al. (2016) proposed that WMC and gF tap different subfunctions of AC. WMC reflects maintenance—the ability to preserve task goals and representations in an accessible, interference-resistant state—whereas gF places stronger demands on disengagement—the ability to abandon misleading information or failed solution paths and explore alternatives. For example, complex span tasks such as operation span prioritize maintenance, while reasoning tasks including Raven’s matrices require flexible disengagement and representational updating. Figure 1 illustrates this framework, showing how executive attention governs both maintenance and disengagement processes in response to task demands. Although these functions play different roles across tasks, they both rely on the broader capacity to control attention. From this perspective, WMC is best viewed as a partial expression of attention control, centered on maintenance, but with residual variance potentially attributable to storage or binding-specific processes (A. Baddeley, 2012; Oberauer, 2009). Recognizing this shared but asymmetrical structure between AC, WMC, and gF is essential for clarifying their respective contributions to individual differences in cognition.

### 1.2. Task-Level Evidence Linking Working Memory Capacity and Attention Control

At the task level, many of the empirical links between WMC and AC arise because standard WMC measures embed substantial attention-control demands. In fact, WMC measures—such as complex span tasks—include both a storage component and an AC component (Engle et al., 1999; Unsworth & Engle, 2007). These tasks usually embed a memory load within a secondary processing demand, requiring the individual to maintain information while resolving interference or updating content—thereby engaging both maintenance and control processes (Conway et al., 2005; Draheim et al., 2022). Consistent with this view, individuals with high WMC tend to perform better on AC tasks that involve minimal memory demands (Conway et al., 2001; Heitz & Engle, 2007; Kane et al., 2001; Kane & Engle, 2003; McVay & Kane, 2009; Unsworth et al., 2004).

Latent-variable studies using structural equation modeling (SEM) reinforce this interpretation, showing that much of what WMC predicts is shared with AC. When the variance shared with AC is statistically controlled, WMC’s links to higher-order criteria often diminish substantially. For example, Engle et al. (1999) showed that WMC predicts gF independently of short-term memory, implicating domain-general executive attention rather than passive storage. This early finding laid the foundation for later distinctions among specific AC subfunctions. Tsukahara et al. (2020) and Draheim et al. (2021) found that AC fully mediated the relationship between WMC and sensory discrimination or gF, respectively, with the tasks indexing interference resolution and attentional disengagement. Multitasking performance relies substantially on the controlled mechanisms underlying WMC, with a mediation analysis showing that capacity and attention control fully account for the WMC–multitasking relationship (Redick et al., 2016), and subsequent findings indicating that a latent AC factor alone can account for the majority of multitasking variance and substantially attenuate the WMC–gF correlation (Burgoyne et al., 2023).

Table 1 summarizes these findings, mapping each study’s outcome, model structure, and the specific AC mechanisms implicated in mediating the effects of WMC.

### 1.3. Reevaluating Working Memory Capacity Research with Attention Control

Despite converging evidence in support of AC, many studies continue to frame cognitive performance in terms of WMC. Draheim et al. (2022) attribute this persistent emphasis to two main factors: historical inertia stemming from WMC’s early success as a broad individual-differences construct, and the psychometric limitations of earlier AC measures, which lacked sufficient reliability and validity until more recently.

Grounded in recent advances in the conceptualization (e.g., Shipstead et al., 2016; Tsukahara et al., 2020) and measurement (e.g., Draheim et al., 2021; J. D. Martin et al., 2021) of AC, Draheim et al. (2022) systematically re-evaluated a wide range of applied domains. They argued that attention control, not WMC, more fundamentally explains performance across diverse settings. These findings also raise the possibility that AC is itself multidimensional, comprising distinct but interacting subfunctions such as goal maintenance, interference resolution, and disengagement (Draheim et al., 2021; Miyake et al., 2000).

In this review, building on such empirical demonstrations, we will deeply interrogate the theoretical assumptions behind WMC-framed findings. We argue that many of these findings are not just empirically better predicted by AC but are more coherently explained by attention-based mechanisms when the cognitive demands of the tasks are examined closely. Clarifying how AC serves as a more central explanatory construct than WMC can inform both theoretical models and real-world applications. The broader implications of this reframing—for assessment, intervention, and applied decision-making—are discussed in later sections.

### 1.4. Current Paper’s Thesis and Structure

This paper challenges the widespread assumption that WMC is a foundational cognitive construct, arguing instead that its predictive power primarily reflects underlying mechanisms of AC. Guided by a theoretically motivated synthesis, we examine various cognitive performance contexts to evaluate the explanatory reach of AC relative to WMC. Our goal is to clarify the mechanisms driving observed individual differences by reframing WMC not as a unitary proxy for ability, but as a composite construct whose predictive value depends on its shared variance with AC. In doing so, we aim to advance a reconceptualization that integrates empirical findings into a more precise and mechanistically grounded framework for cognitive performance.

## 2. Domain-Based Re-Evaluation of WMC and Attentional Control

To assess how well AC accounts for findings commonly attributed to WMC, we examine six cognitive domains where WMC has frequently served as a primary explanatory construct. These include (1) perception, sensory discrimination, and early processing (2) reasoning, problem-solving, and academic performance, (3) decision-making, self-control, and cognitive control performance, (4) retrieval and memory, (5) multitasking and real-world task performance, and (6) clinical dysfunctions. While WMC has often been used to predict performance in these contexts, we consider whether closer inspection of task demands and mechanisms suggests a more central role for AC processes—such as goal maintenance, interference resolution, and attentional disengagement—in shaping individual differences.

### 2.1. Perception, Sensory Discrimination, and Early Processing

Perceptual success—whether in detecting relevant stimuli, discriminating sensory input, or maintaining visual focus—is often attributed to differences in WMC (Allen et al., 2011; Bayramova et al., 2021; Hughes et al., 2013; Sorqvist, 2010; Yurgil & Golob, 2013). However, rather than reflecting enhanced storage alone, these effects may also stem from individuals’ ability to exert AC over competing sensory inputs. Desimone and Duncan’s (1995) biased competition framework proposes that attention resolves competition among stimuli by biasing processing toward task-relevant inputs. This bias is guided by selection templates maintained in working memory (A. Baddeley et al., 1986; Desimone & Duncan, 1995) and is neurally implemented via top-down signals from prefrontal and parietal regions that modulate sensory cortex activity. This model offers a compelling account of how AC shapes sensory processing (Awh & Jonides, 2001; Gazzaley & Nobre, 2012).

Gazzaley and Nobre’s (2012) review further elaborates these mechanisms by specifying how prefrontal control regions dynamically modulate visual cortex to prioritize goal-relevant information. EEG studies have shown that attentional modulation of visual cortical activity during early encoding predicts later memory performance (Rutman et al., 2010), and fMRI research demonstrates that functional coupling between prefrontal areas and visual regions enhances relevant signals while suppressing distractors (Chadick & Gazzaley, 2011; Gazzaley et al., 2007). Consistent with this, high-WMC individuals deploy visual attention more flexibly and precisely (Bleckley et al., 2003), while variation in visual working memory appears to reflect differences in attentional filtering rather than pure storage limits (Luck & Vogel, 2013), particularly under high-interference conditions (Ester et al., 2014; Soto et al., 2010).

Auditory evidence also highlights AC. High-WMC individuals show reduced early brain responses to irrelevant inputs, consistent with stronger sensory gating (Sörqvist et al., 2012; Tsuchida et al., 2012). However, not all forms of distraction are equally susceptible to control. WMC predicts resistance to distraction effects that require top-down control (e.g., deviation effect) but not those driven by automatic interference (e.g., changing-state sequences) suggesting that attentional mechanisms play a role only when distraction is controllable (Hughes et al., 2013; Sorqvist, 2010). The cocktail party effect illustrates this well: low-WMC individuals are more often distracted by automatically salient stimuli such as their name in an unattended auditory stream, indicating a failure to suppress irrelevant input (Conway et al., 2001). When the task requires divided monitoring, however, high-WMC individuals are more likely to detect their name, demonstrating flexible attention deployment (Colflesh & Conway, 2007).

While these studies often rely on WMC measures, many describe AC as the operative mechanism. Some openly frame WMC as reflecting filtering or goal maintenance function distraction (Hughes et al., 2013; Sorqvist, 2010), and others invoke attentional selection when explaining perceptual stability, as in the case of bistable figures (Allen et al., 2011). Even studies that more strongly emphasize WMC as the underlying construct—such as those showing enhanced sensory gating under high cognitive load or reduced distractor-evoked ERP responses—may be reinterpreted as reflecting more efficient attentional suppression (Bayramova et al., 2021; Yurgil & Golob, 2013). Importantly, recent findings suggest that AC significantly accounts for the association between sensory discrimination and intelligence, though the extent of mediation depends on task and model specification (Tsukahara et al., 2020).

Overall, while storage-based accounts should not be dismissed, the bulk of evidence indicates that attentional selection, filtering, and suppression mechanisms play a primary role in perceptual performance under interference. Apparent WMC advantages in perception may therefore reflect the engagement of top-down AC processes rather than enhanced storage capacity per se.

### 2.2. Reasoning, Problem-Solving, and Academic Performance

WMC is frequently cited as a key predictor of an individual’s ability to reason effectively, solve problems, and succeed academically (Cowan, 2014). Numerous studies associate WMC with outcomes in tasks such as problem-solving (Beilock & DeCaro, 2007; Copeland & Radvansky, 2004; Seyler et al., 2003), reading comprehension (Bayliss et al., 2003; Carretti et al., 2009), achievement in science and mathematics (Giofrè et al., 2018; Musso et al., 2019), and other academic success across domains (Bull & Scerif, 2001). However, individual differences in WMC are often interpreted as reflecting variation in AC components. For example, Whitebread (1999) found that the relationship between WMC and children’s performance was dependent on their metacognitive abilities and strategy use, and Wang and Kao (2022) showed that while both WMC and self-regulated learning (SRL) were significant independent predictors of academic achievement, SRL exhibited the largest effect.

Still, a subset of studies emphasize the role of storage capacity in reasoning and academic performance (Buehner et al., 2005, 2006; De Smedt et al., 2004; Fung & Swanson, 2017; Krumm et al., 2009). For example, Buehner et al. (2005) found that storage consistently predicted reasoning outcomes, a finding replicated in their later work (Buehner et al., 2006) and by Krumm et al. (2009), who concluded that short-term storage, rather than executive components, accounted for the WMC-reasoning link. Fung and Swanson (2017) similarly reported that the phonological loop directly predicted math problem-solving accuracy. However, a closer look at these studies suggests their conclusions may overstate the independence of storage from control processes. First, Buehner’s attention measures focused narrowly on selectivity, omitting critical facets of AC such as goal maintenance and interference resolution. Without capturing these mechanisms, it is difficult to conclude that attention per se is unrelated to reasoning. Second, Buehner and Krumm’s definition of storage as “storage in the context of processing” implicitly involves coordination and executive oversight, making the construct more aligned with controlled maintenance than with passive retention. Third, the coordination factor emphasized by Krumm—later refined as relational integration by Oberauer et al. (2008)—requires executive-level binding of multiple representations, a process that reflects domain-general AC. Finally, Fung and Swanson (2017) used executive measures (e.g., conceptual span), which may have suffered from task impurity, blending executive and knowledge-based demands. These limitations caution against interpreting such findings as evidence that storage alone underlies reasoning and instead support the view that AC remains central to understanding the predictive power of WMC in academic and reasoning contexts.

A broader perspective situates reasoning and academic performance within fluid intelligence, highlighting their shared reliance on attention control mechanisms. Reasoning (Carroll, 1993; Kyllonen & Christal, 1990; Sternberg, 1986), problem-solving (Duncan et al., 2008) have been widely accepted as proxies for gF (Kane et al., 2005; Primi, 2002) along with academic achievement (Ackerman & Heggestad, 1997; Farsides & Woodfield, 2003). The observed associations between gf and WMC (Conway et al., 2002; Jaeggi et al., 2008; Jastrzebski et al., 2018; Kane et al., 2005) likely reflect their shared dependence on AC mechanisms rather than separate structural capacities (Engle et al., 1999; Shipstead et al., 2016). Duncan et al. (2012) demonstrated that gF closely aligns with performance on rule-based working memory tasks, which involve constructing and managing novel representations—tasks that rely heavily on AC—rather than simpler storage tasks such as digit span, spatial span, or visual short-term memory. Hambrick and Engle (2003) similarly found that shared variance between WMC and reasoning tasks was best explained by AC processes.

Numerous studies have interpreted reasoning, problem-solving, and academic achievement through the lens of executive functioning (EF) (e.g., García-Madruga et al., 2022; Latzman et al., 2010; Miyake et al., 2000; Spiegel et al., 2021; Willoughby et al., 2012). EF is traditionally characterized by three core components—updating, shifting, and inhibition (Miyake et al., 2000), which can be mapped onto the maintenance–disengagement distinction emphasized in later AC frameworks (Engle, 2002; Kane & Engle, 2003; Shipstead et al., 2016). Goal maintenance corresponds to keeping task-relevant representations active, a process that overlaps with updating when defined as controlled refreshing and with aspects of inhibition when it supports protection of the current goal state (García-Madruga et al., 2022; Oberauer et al., 2003). In contrast, disengagement reflects the deliberate dropping or clearing of no-longer-useful information, aligning with the shifting component of EF and with the substitution aspect of updating—both of which require releasing outdated representations so new ones can be instantiated.

Meanwhile, dual-process theory offers insight into how AC supports reasoning. It distinguishes System 1, which is fast and automatic, from System 2, which is deliberate and effortful (Evans, 2003; Evans & Stanovich, 2013; García-Madruga et al., 2007, 2022; Kahneman, 2011; Sloman, 1996). Sustained AC underlies System 2’s ability to override heuristics and construct valid representations. High-WMC individuals, who exhibit greater AC, tend to favor rule-based strategies and outperform others on deductive reasoning tasks (Beilock & DeCaro, 2007; García-Madruga et al., 2007; Wiley & Jarosz, 2012). However, the same sustained control can also lead to rigidity or overfocus, contributing to underperformance in creative contexts or under pressure (Beilock & DeCaro, 2007; Wiley & Jarosz, 2012). These findings illustrate that AC can both support and constrain reasoning, depending on the demands for flexibility versus stability.

### 2.3. Decision-Making, Self-Control, and Cognitive Control Performance

Building on this dual-process perspective, decision-making and self-control performance rely on cognitive control operations grounded in attention control. Defined by attentional functions such as goal maintenance and inhibition, cognitive control embodies the effortful regulation required to guide behavior in line with goals (Braver, 2012; Redick, 2014). Although studies often use WMC tasks to measure ‘control’ as the counterpart to automatic/associative processing, the construct is implemented by AC operations—maintaining goals, prioritizing task-relevant information, and suppressing prepotent/automatic responses (Barrett et al., 2004; Fletcher et al., 2011). Braver et al.’s (2007) dual mechanisms of control framework further distinguishes proactive control (sustained, anticipatory goal maintenance) and reactive control (phasic, interference-triggered reconfiguration), both grounded in attentional regulation.

However, such control can carry context-dependent costs. In risky-choice framing, high-WMC individuals showed greater susceptibility to framing effects, likely due to elaborate, gist-based encoding (Corbin et al., 2010). This tension is pronounced in the AX Continuous Performance Task (AX-CPT), which probes control dynamics with sequences of cue–probe pairs (e.g., “Respond to ‘X’ only if it is preceded by ‘A’). Proactive control facilitates performance for the target cue–probe pairs. However, target response proactively prepared after ‘A’ must be inhibited when the interfering ‘Y’ follows, producing slower and more error-prone responses. High-WMC individuals often show larger interference costs in these trials, but this may reflect strategic prioritization of proactive control rather than an attentional failure (Redick, 2014). If WMC primarily reflected storage capacity, we would expect uniformly better performance across trial types. Instead, the variability in success across proactive- and reactive-reliant conditions underscores that what WMC measures were predicting the ability to regulate and deploy attention in alignment with contextual goals instead (however, Rosales et al., 2022 argue that high-WMC individuals generally perform well across both proactive and reactive, still interpreting it as reflecting overall control efficiency).

These mechanisms of cognitive control extend into everyday decision contexts. In morally and ethically complex decisions, high-WMC individuals tend to favor utilitarian judgments and perform better in ethical reasoning tasks because they are more likely to suppress affective interference, sustain abstract goal states, and coordinate multiple competing constraints—operations grounded in executive deliberation and attentional filtering (A. Martin et al., 2015; Moore et al., 2008). In tactical sports contexts, AC enables athletes to override prepotent, irrelevant responses and adapt dynamically to situational demands (Furley & Memmert, 2012), while in syllogistic reasoning tasks, WMC predicts better performance via attentional suppression of biases and focus on goal-relevant stimuli (Fletcher et al., 2011). In high-demand decision contexts involving uncertainty or competing goals, AC facilitates consistent goal pursuit and suppression of irrelevant reactivity (Rakow et al., 2010), and WMC facilitates goal fulfillment such as achieving cognitive closure quickly (Czernatowicz-Kukuczka et al., 2014).

Neuroscientific evidence reinforces the view that attention control underlies cognitive control. The prefrontal cortex (PFC), particularly the lateral regions, provides the substrate for sustaining goals and resolving interference, flexibly coordinating thought and action (Miller & Cohen, 2001). Within the Dual Mechanisms of Control framework, sustained PFC activation supports proactive control, while transient responses reflect reactive engagement—mirroring attentional dynamics (Braver, 2012). Supporting this, dissociations with posterior regions show that distractor resistance and flexibility rely on recurrent PFC circuitry rather than storage-based representations (Murray et al., 2017). Also, High-WMC individuals exhibit increased activation in frontoparietal regions associated with AC—particularly the dorsolateral and anterior prefrontal cortex—during tasks requiring sustained goal maintenance and conflict resolution (Jimura et al., 2018; Minamoto et al., 2015). These neural patterns are consistent with AC, though PFC activation may also reflect broader cognitive operations such as conflict monitoring or strategic retrieval.

### 2.4. Retrieval and Memory

Although strongly related to WMC, many retrieval advantages in fact hinge on AC, particularly the ability to inhibit competing information. Defined as suppressing irrelevant responses (Aslan & Bauml, 2011; Engle et al., 1999), inhibitory control is effectively utilized by High-WMC individuals to perform better on retrieval tasks. For instance, Rosen and Engle (1997) reported that high-WMC participants suppressed first-list responses under interference conditions in a paired-associates task. Related work on verbal fluency has shown that high-WMC individuals are better at suppressing previously relevant but currently irrelevant responses; however, this effect has been reevaluated as potentially confounded by gF, with follow-up analyses suggesting that performance is better accounted for by individual differences in gF (Shipstead et al., 2016).

Other work has shown that high-WMC individuals commit more retrieval-induced forgetting (RIF) (Aslan & Bauml, 2011)—the tendency to forget unpracticed items from a studied category after repeatedly retrieving related items—and more pronounced negative priming effects under high-interference conditions (Marsh et al., 2015), both reflecting executive inhibitory engagement. However, divergent findings exist. For example, Mall and Morey (2013) found that high-WMC individuals showed less RIF. This discrepancy has been attributed to task format differences: while Aslan and Bäuml used a short-delay item recognition test that tapped suppression processes during encoding and short-term retrieval, Mall and Morey used a cued recall task with a 25 min delay, emphasizing retrieval from long-term memory. The latter relied more on targeted search and focused cue access—mechanisms associated with controlled search from secondary memory rather than active inhibition (Kliegl & Bäuml, 2021). More broadly, research supports the view that retrieval reflects both AC and memory-intrinsic mechanisms, such as representational strength, trace competition, and cue-dependence. Controlled attention is crucial for minimizing interference during encoding, maintaining task goals, and suppressing competing traces at retrieval (Hamilton et al., 2022; Kotyusov et al., 2023; Unsworth & Engle, 2007). At the same time, retrieval success also depends on cue quality, search dynamics, and the stability of stored representations, especially under conditions of high similarity or delay (Kliegl & Bäuml, 2021).

One thing to note is that inhibition is more closely associated with gF than WMC (Shipstead et al., 2016). In fact, some studies report positive correlations between gF and retrieval performance (e.g., de Lima & Buratto, 2025; Minear et al., 2018). As mentioned in the reasoning, problem-solving, and academic performance section, WMC and gF are closely related constructs (Conway et al., 2002; Jaeggi et al., 2008; Jastrzebski et al., 2018; Kane et al., 2005), largely due to their shared reliance on a top-down executive attention system (Engle et al., 1999; Shipstead et al., 2016). Accordingly, inhibition in high-WMC individuals during retrieval tasks likely reflects overlapping attentional mechanisms common to both WMC and gF, though the degree and direction of influence may vary across tasks.

Such a pattern, in fact, manifests across each stage of memory. During encoding, WMC benefits emerge from active organizational strategies, prioritization of valuable information, and distraction suppression (Robison & Unsworth, 2017; Weeks & Hasher, 2017). During retrieval, differences in WMC are pronounced in tasks requiring interference resolution, strategic cue access, or selective recall (Rosen & Engle, 1997; Unsworth et al., 2012). Even at the storage stage, WMC reflects how attention consolidates perceptual inputs into stable traces (Ricker et al., 2018) and protects them via refreshing, interference removal, or strategic off-loading into activated long-term memory (Rhodes & Cowan, 2018).

In sum, memory retrieval does not operate independently of AC but is largely shaped by them—whether through direct inhibition or strategic guidance of search. WMC serves as an index of how flexibly attention can be deployed across memory demands. Nonetheless, memory-intrinsic dynamics also play a critical role, and task format, cue structure, and delay intervals can shift reliance between AC and storage-based retrieval processes. Understanding this interplay highlights the role of AC on goal-directed memory performance.

### 2.5. Multitasking and Real-World Task Performance

To generalize laboratory findings to real-world cognition, multitasking provides an effective starting point: it requires managing competing goals, coordinating responses across modalities, and adjusting plans in real time. While WMC is often treated as the primary predictor in these contexts (Colom et al., 2010; Hambrick et al., 2010; Konig et al., 2005; Redick, 2016; Redick et al., 2016), evidence shows that success depends less on storage and more on regulating task goals, suppressing distraction, and reallocating resources—functions of AC.

Himi et al. (2023) reviewed 43 studies on individual differences in multitasking and showed that although WMC often emerged as a predictor, its unique contribution diminished when shared variance with executive functions was modeled. In contrast, higher-level control abilities—such as updating and relational integration—remained robust and theoretically central predictors of multitasking performance (Friedman et al., 2008, 2016; Himi et al., 2019; see also Chuderski, 2014, arguing that relational integration functions independently of significant executive control). These findings suggest that successful multitasking relies less on storage capacity per se and more on AC operations that support goal maintenance and the suppression of distraction.

Likewise, studies linking WMC to multitasking often highlight its underlying attentional mechanisms rather than storage (Engle et al., 1999; Kane et al., 2007). Otermans et al. (2022) showed that performance drops under dual-task or order-change conditions were better explained by goal sequencing and interference control—both AC mechanisms—than by storage limitations. In applied simulations like SynWin, performance was predicted by executive attention and strategic allocation rather than raw capacity, with single-task and complex-span measures contributing similarly (Hambrick et al., 2010; Redick, 2016) extended these findings to more complex environments, including Air Traffic Control scenarios, showing that the relationship between WMC and multitasking was fully accounted for by capacity and attention control, alongside related executive processes. This pattern reinforces the view that WMC predicts multitasking largely because of its shared variance with AC mechanisms, rather than through storage-specific pathways. Driving studies also trace distraction-related costs to goal maintenance and inhibitory control (Louie & Mouloua, 2019; Mäntylä et al., 2009; Wood et al., 2016).

Real-world interruptions follow the same logic. In safety-critical settings, interruptions often cause errors (Foroughi et al., 2014; Latorella, 1996; Westbrook et al., 2018). Some studies have linked resistance to such interruptions to the inhibitory function of AC (Mirhoseini et al., 2020; Tams et al., 2015) as reviewed in Draheim et al.’s (2022) work. A similar pattern emerges in laboratory simulations of everyday multitasking. The Edinburgh Virtual Errands Test (EVET) requires participants to navigate a 3-D environment while managing multiple errands. Performance in EVET is predicted not by verbal storage or rehearsal, but by AC-related processes such as spatial attention, goal monitoring, and updating (Law et al., 2013; Logie et al., 2011).

Neuroscientific and computational modeling evidence further support this interpretation. For example, Medeiros-Ward et al. (2015) showed that “Supertaskers” more efficiently recruited the anterior cingulate and frontopolar cortices—regions critical for goal maintenance and distraction suppression—during a demanding dual N-back task, with reduced medial PFC activation at high loads indicating neural efficiency. Complementing this, Nijboer et al. (2016) showed that multitasking interference reflects resource competition within a distributed working memory system, and that models including control-related components fit behavioral and fMRI data better than capacity-only accounts. Although these studies do not directly compare the neural substrates of WMC and AC, the patterns are compatible with the view that multitasking engages domain-general control systems and that AC-related mechanisms contribute meaningfully to performance.

Taken together, these behavioral, computational, and neural findings suggest that many effects historically attributed to WMC in multitasking contexts can also be understood through the lens of AC. Across structured simulations and immersive environments, multitasking success appears to depend on the regulation of goals, resistance to interference, and the flexible coordination of cognitive operations—while acknowledging that multiple mechanisms beyond AC may also play supportive roles.—while leaving room for contributions from strategy, knowledge, and processing speed without overextending the explanatory scope of any single construct.

### 2.6. Clinical Dysfunctions

Internalizing and externalizing disorders are widely understood to involve cognitive and/or AC deficits, including ADHD (Barkley, 1997; Nigg, 2000, 2001), depression (Gotlib & Joormann, 2010; Joormann & Gotlib, 2008; Keller et al., 2019), anxiety (Derryberry & Rothbart, 1997; Reinholdt-Dunne et al., 2009), and schizophrenia (Barch, 2005; Luck & Gold, 2008). Reframing these dysfunctions through the lens of AC provides a coherent way to understand why similar patterns of cognitive dysregulation appear across otherwise distinct disorders. This perspective does not deny the importance of motivational, affective, neurobiological, or representational factors; rather, it highlights how these processes often interact with difficulties in attentional regulation. The aim is therefore to clarify AC’s role as a meaningful contributor—without suggesting it is the sole basis—while keeping neural and mechanistic interpretations appropriately cautious.

#### 2.6.1. ADHD

ADHD inherently implicates attentional and inhibitory deficits, and foundational models frame the disorder as involving failures of behavioral inhibition and attentional regulation (Barkley, 1997; Nigg, 2000, 2001; Pliszka et al., 2000). Barkley’s (1997) seminal theory identifies ADHD as rooted in primary deficits in behavioral inhibition, which give rise to broader executive dysfunctions. Kofler et al. (2010) similarly found that central executive deficits, particularly those linked to AC, predicted inattentive behavior.

Still, ADHD research often interprets impairments in terms of WMC. Meta-analytics by Martinussen et al. (2005), for instance, shows reduced performance on WMC tasks, though many of these tasks embed attentional demands. Dual-component models by Gibson et al. (2010, 2018) report greater deficits in secondary memory retrieval, which can be partially reinterpreted as failures of attentional gating—difficulty sustaining access to goal-relevant information. Neuroimaging findings align with this interpretation but require caution: reduced frontoparietal activation in adults with ADHD (Burgess et al., 2010) may index impaired goal maintenance yet could also reflect broader cortical inefficiency or reduced task engagement. Luo et al. (2019) likewise reported attenuated ERP markers of selection (reduced N2pc) and maintenance (reduced CDA), consistent with reduced efficiency in attentional selection and working memory processes, which they interpret as evidence that reduced working memory performance is closely tied to insufficient attentional selection ability. Their findings highlight a specific selection-related mechanism without addressing alternative explanations such as broader cortical inefficiency or reduced task engagement.

At the same time, ADHD symptoms reflect contributions from motivational dysregulation, arousal variability, and delay aversion (Barkley, 1997), as well as difficulties in sustaining stable representational states (Barkley, 1997; Gibson et al., 2010, 2018). These factors interact with—but are not reducible to—attentional regulation, consistent with models positioning poor sustained attention as a secondary outcome of broader executive and motivational impairments.

#### 2.6.2. Affective Disorders

Affective disorders frequently involve heightened distractibility, difficulty disengaging attention from negative stimuli, and reduced cognitive flexibility—symptoms tied to AC limitations (Derryberry & Rothbart, 1997; Harvey et al., 2004; Rothbart et al., 2004). Attentional Control Theory (ATC; Derakshan & Eysenck, 2009; Eysenck et al., 2007) describes how anxiety undermines inhibitory control and shifting, especially under load. Depression likewise involves slowed attentional disengagement and rumination-driven narrowing of attentional scope (Whitmer & Gotlib, 2013; Yaroslavsky et al., 2019). Although AC deficits relate closely to symptom severity, these associations are bidirectional and occur alongside mood-congruent processing biases, emotional reactivity, and representational disturbances. In this context, attentional processes operate in interaction with affective mechanisms that are central features of anxiety and depression, indicating that AC is one important pathway influencing symptom expression but not the only one.

Meta-analytic work supports AC as an important predictor of anxiety. Moran (2016) showed that anxiety’s association with WMC (g = −0.33) was modest compared to filtering efficiency (g = −0.70), though differences in task properties and sample characteristics caution against overinterpreting effect-size contrasts. In another review, Shi et al. (2019) similarly reported strong links between AC and anxiety (g = −0.58). Even research framed around WMC in depression often explains deficits through AC—failures to inhibit irrelevant negative content or maintain goal-relevant representations (Joormann & D’Avanzato, 2010; Joormann & Gotlib, 2008). Intervention studies further support this interpretation: mindfulness and adaptive WMC training improve symptoms partly by enhancing AC (Beloe & Derakshan, 2020; Vago & Silbersweig, 2012). Still, these improvements likely arise from multiple mechanisms, including reduced emotional reactivity and enhanced representational stability, yet they also highlight that strengthening attentional regulation can play a meaningful role in alleviating affective symptoms.

#### 2.6.3. Schizophrenia

Historical and contemporary accounts of schizophrenia describe pervasive impairments in attentional regulation, including difficulties with rule selection and context processing (Bleuler, 1911/1950; Kraepelin, 1919). Inhibitory deficits become especially pronounced under high attentional load, consistent with evidence that increased demands strain both attentional resources and inhibitory control (Henik & Salo, 2004). Some findings further suggest that these difficulties reflect not only resource limitations but also failures of inhibition itself (Michael et al., 2020). Within this framework, WMC supports the maintenance and implementation of task rules but is often overshadowed by failures in attentional regulation.

At the same time, schizophrenia research frequently interprets cognitive deficits as WMC impairments—difficulties in maintaining and manipulating information (Barch, 2005; Gold et al., 1997). Yet these deficits are often described in terms of disrupted context processing, impaired attentional filtering, and limited inhibitory resources (Barch, 2005; Michael et al., 2014), all closely tied to AC. Nevertheless, schizophrenia also involves representational and sensory-processing abnormalities, such as deficits in perceptual organization, N100/P50 sensory gating, and contextual integration, which extend beyond attentional regulation and highlight opportunities to examine how these perceptual and contextual disturbances interact with AC.

Across clinical populations, cognitive impairments often attributed to WMC reflect, at least in part, difficulties in regulating attention. However, ADHD, affective disorders, and schizophrenia each exhibit distinct cognitive signatures shaped by motivational, representational, affective, and neurobiological factors. Attentional control therefore provides a unifying—although not exhaustive—framework: a major contributor that interacts with multiple disorder-specific mechanisms.

## 3. Discussion

This review examined six domains to determine when effects attributed to WMC instead reflect AC. By comparing task demands across perception, learning and problem-solving, reasoning, decision-making, retrieval, multitasking, and clinical outcomes, we identified where “capacity” effects arise from control operations such as goal maintenance, interference suppression, and disengagement. Building on the direction outlined by Draheim et al. (2022) and Pak et al. (2024), this review advances the WMC–AC framework by (a) offering a deeper theoretical articulation of how core AC mechanisms—goal maintenance, interference suppression, and disengagement—relate to patterns traditionally associated with WMC, and (b) demonstrating, across six comprehensive cognitive domains, how this refined articulation generalizes to diverse findings through a consistent, mechanism-based interpretation.

### 3.1. Task-Level Measurement: Dissociating AC and WMC

Going forward, research should rely more directly on attention-control measures instead of relying on complex span as a proxy. Complex span variants (e.g., operation, rotation, symmetry) are primary WMC indices, yet their broad predictiveness arises because the paradigm embeds control demands: processing and to-be-remembered items are interleaved and followed by serial recall, so success depends on stabilizing actively maintaining the goal state during interference and distraction, continuously updating information, and preventing intrusions from irrelevant or automatically activated information (Conway et al., 2001; Engle et al., 1999; Giofrè et al., 2018; Miyake et al., 2000; Swanson & Alloway, 2012; Yurgil & Golob, 2013); by contrast, simple span tasks minimizes interference and correspondingly predicts more weakly (Conway et al., 2002; Engle, 2002). Visual-arrays tasks—especially selective/filtering versions—converge on the same point: once effective filtering is cued or supported, apparent performance gaps contract, identifying selection as the operative bottleneck (Tsukahara et al., 2020). The same control variance is evident in classic AC tasks such as antisaccade, Stroop, and flanker, which require suppressing prepotent responses and keeping rules active under conflict with minimal mnemonic load (Engle, 2002; Kane & Engle, 2003; Kane et al., 2006; Miyake et al., 2000; Tsukahara et al., 2020). Continuous updating paradigms like the n-back, selective attention under competition (e.g., dichotic listening), and task switching likewise depend on restricting access to outdated representations and disengaging from prior sets on time. Accordingly, future studies should include high-reliability AC measures (e.g., Squared Flanker, Squared Simon, and Squared Stroop; Burgoyne et al., 2023) alongside well-designed WMC tasks that separately manipulate maintenance and interference demands. Orthogonally varying set size, distractor salience, parallel processing, and filtering cues—rather than embedding all demands within a single composite score—will allow clearer identification of the specific control operations that limit performance. This task-level refinement is a necessary foundation for distinguishing storage from control processes and for improving construct validity moving forward.

### 3.2. Latent-Variable Approaches

Although WMC and AC can each be decomposed into more fine-grained operations—such as goal maintenance, interference suppression, and disengagement—current measurement science offers no process-pure tasks capable of isolating these components with adequate reliability. Most laboratory tasks confound multiple operations (the “task impurity problem”; Miyake et al., 2000), making single-task estimates too noisy to serve as direct indicators of individual processes. Construct-level latent variables therefore remain essential because they capture the covariance shared across tasks, separating the common control component from task-specific variance. In this framework, AC and WMC function as empirically tractable clusters of underlying mechanisms, allowing those mechanisms to be dissociated statistically even when they cannot be measured in isolation. Process-level analyses remain theoretically informative, but latent-variable modeling currently provides the most stable and interpretable method for distinguishing storage, control, and representational contributions to cognitive performance.

Such value of latent-variable modeling is demonstrated across multiple lines of work. Classic working-memory studies (Engle et al., 1999; Conway et al., 2002) demonstrated that latent WMC predicts gF beyond short-term storage. Executive-function models similarly separate common control processes from shifting- and updating-specific variance (Miyake et al., 2000; Friedman et al., 2008, 2016). Dual-component accounts further break WMC into attention-control and secondary-memory components (Unsworth & Spillers, 2010), and more recent SEM work isolates AC as a separable latent factor that accounts for substantial variance in gF, WMC, and multitasking (Tsukahara et al., 2020; Draheim et al., 2021; Burgoyne et al., 2023). Together, these findings show that latent modeling provides a principled route for clarifying when WMC and AC converge and when their underlying mechanisms diverge.

## 4. Conclusions

Across six domains, the evidence reviewed here suggests that the broad predictive power traditionally associated with WMC often reflects the AC operations embedded within complex-span tasks—particularly goal maintenance, interference suppression, and disengagement. This does not diminish the importance of WMC as a measurable construct; rather, it clarifies that many WMC tasks draw on AC mechanisms, which are more directly tied to performance in interference-heavy contexts. With recent advances in high-reliability AC measures, it becomes possible to assess these mechanisms more directly and with greater precision than was feasible when WMC tasks served as the default proxy for executive attention.

This shift toward measuring AC more directly also carries practical implications across applied settings. In personnel selection, AC-based assessments show smaller subgroup differences and less dependence on acculturated knowledge than many traditional cognitive tests, suggesting a fairer and more targeted basis for evaluating complex task readiness (Bosco et al., 2015; Burgoyne et al., 2021). In high-stakes environments—such as policing, aviation, and other dynamic decision contexts—AC predicts operators’ ability to maintain goals, adjust criteria, and override prepotent responses under pressure (Brewer et al., 2016; Kleider & Parrott, 2009; McVay & Kane, 2009). Clinical and educational findings similarly point to attentional regulation as a closer determinant of symptoms and learning difficulties than storage capacity alone, with interventions improving outcomes largely through strengthened control processes (Dehn, 2011; Draheim et al., 2022; Moran, 2016).

## Figures and Tables

**Table 1:** Summary of latent-variable studies investigating the predictive power of attention control (AC) for the relationship between working memory capacity (WMC) and other cognitive abilities.

Study (Author, Year)	Latent Model Structure	Effect of Controlling for AC	Primary AC Mechanism Involved
Engle et al. (1999)	Latent WMC (Complex Span tasks) and Latent STM predicting Latent gF. The AC component was indexed by the WMC residual after controlling for STM.	WMC (residual AC component) still significantly predicted gF (β = 0.49). STM did not predict gF, highlighting the role of executive attention.	General Executive Attention/Controlled Attention (maintaining representations in the face of interference).
Tsukahara et al. (2020)	Latent AC factor (accuracy-based tasks) mediating the relationship between Latent WMC and Latent Sensory Discrimination.	AC fully mediated the WMC–Sensory Discrimination relationship. WMC no longer statistically significant.	Interference Resolution/Suppression and Attentional Disengagement.
Draheim et al. (2021)	Latent AC factor (reliable accuracy-based tasks) mediating the relationship between Latent WMC and Latent gF.	AC fully mediated the WMC–gF relationship. WMC was no longer statistically significant.	Attentional Disengagement/Shifting and Interference Resolution.
Redick et al. (2016)	WMC predicts Latent Multitasking via Capacity and AC latent factors.	Capacity and AC fully mediated WMC–Multitasking relationship. WMC direct path not significant.	Goal Maintenance and Interference Resolution/Filtering.
Burgoyne et al. (2023)	Latent AC (Squared conflict tasks) predicting Latent WMC, gF, and Multitasking.	AC accounted for 75.6% of Multitasking variance and reduced WMC–gF correlation from r = 0.63 to r = 0.40.	Goal Maintenance and Interference Resolution/Suppression.

## Data Availability

No new data were created or analyzed in this study.

## Abbreviations

The following abbreviations are used in this manuscript:

WMC	Working memory capacity
AC	Attention control
gF	Fluid intelligence
